# Microglial mechanisms of viable retinal ganglion cell elimination

**DOI:** 10.3389/fncel.2025.1719791

**Published:** 2025-12-05

**Authors:** Navita N. López, Yésica Landaverde Rodríguez, Monica L. Vetter

**Affiliations:** Department of Neurobiology, University of Utah, Salt Lake City, UT, United States

**Keywords:** retina, phagocytosis, MERTK, complement receptor 3, CR3, microglia

## Abstract

Microglia can selectively phagocytose live neurons during normal development and also in response to stress, injury or disease by recognizing phagocytic cues to target cells for elimination. In the developing retina at embryonic stages we previously found that microglia refine retinal ganglion cell (RGC) numbers by targeting non-apoptotic newborn RGCs for phagocytosis, utilizing complement receptor 3 (CR3) to recognize and eliminate RGCs. Here, we investigate additional phagocytic mechanisms and cues that microglia utilize to clear a subset of viable RGCs. Our findings indicate that both Mer tyrosine kinase (Mertk) and CR3 are required for clearance of a subpopulation of embryonic RGCs. In Mertk/CR3 double knockouts, we show that C1q-tagged RGCs accumulate and excess RGCs persist indicating failure of normal clearance by microglia. We also show that microglia target RGCs that have phosphorylated c-JUN (p-cJUN) expression, suggesting stress pathway activation. RGCs with p-cJUN expression also accumulate in Mertk/CR3 double knockout retinas, but this appears to resolve by P0, suggesting this is a transient stress state exhibited by a subset of RGCs that remain viable. By depleting microglia we establish that microglia are not required for p-cJUN induction in RGCs but show that they are the sole source of complement protein C1q, which marks these cells for elimination. Altogether the data suggests that a subset of stressed RGCs are recognized by local microglia that tag them with opsonins for removal using specific recognition receptors.

## Introduction

Microglia play well-established roles in phagocytosing damaged or apoptotic neurons in response to stress, injury or disease. However, they also play related roles during normal development of the nervous system, providing an opportunity to define core pathways involved in neuron elimination. Development of the retina requires a delicate balance between neuron production and elimination to attain the appropriate number of cells. During development retinal ganglion cells (RGCs) are produced in excess, and their number is subsequently refined though two waves of elimination, which in mouse occur during embryonic and postnatal periods (Farah and Easter, 2005; Anderson et al., 2019b; Farah, 2006; Péquignot et al., 2003). Resident retinal microglia play distinct roles during these two phases: they influence the survival of RGCs in embryonic development and subsequently mediate the clearance of apoptotic RGCs in postnatal development (Anderson et al., 2019b; Anderson et al., 2022; Braunger et al., 2014). The mechanisms of postnatal RGC apoptosis are influenced by activity and neurotrophic factors (Farah, 2006; Péquignot et al., 2003; Perry et al., 1983; Oppenheim, 1991), while in the embryonic retina apoptosis is minimal and we previously showed that microglia instead engulf living, non-apoptotic RGCs, a process known as primary phagocytosis or phagoptosis (Anderson et al., 2019b; Brown and Neher, 2012). We previously found that microglia directly contact newborn RGCs and form phagocytic cups to ensheathe cleaved caspase 3 (CC3) negative RGCs (non-apoptotic) (Anderson et al., 2019b). The elimination of viable, non-apoptotic neurons by microglia has been documented *in vitro* (Brown and Neher, 2012; Brown and Neher, 2014) and *in vivo* in cortical development, ischemic stroke, and photoreceptor disease (Zhao et al., 2015; Alawieh et al., 2018). Developmental retinal astrocytes and cortical progenitors have also been shown to refine their population through non-apoptotic mechanisms (Puñal et al., 2019; Cunningham et al., 2013). However, the molecular pathways involved in viable cell elimination are only partially defined, particularly *in vivo*.

We previously found that complement signaling is, in part, required for normal elimination of embryonic RGCs by microglia (Anderson et al., 2019b). During the peak period of embryonic elimination, a subpopulation of RGCs is tagged with complement protein C1q, an opsonin that signals via complement receptor 3 (CR3) expressed by microglia. We further showed that disruption of this pathway affects RGC elimination, with ablation of Cd11b (a subunit of CR3) resulting in a 10% excess in RGCs at birth (P0) (Anderson et al., 2019b). Complement signaling has also been implicated in cortical neuron elimination during development and following ischemic stroke (Alawieh et al., 2018; Deivasigamani et al., 2023). However, it is likely that other phagocytic pathways contribute to RGC elimination because depletion of microglia resulted in a larger excess RGC density at birth (~20%) (Anderson et al., 2019b). The TAM receptor tyrosine kinases (Tyro3, Axl, Mertk), are phagocytic receptors that are necessary for clearance of apoptotic cells in other contexts (Scott et al., 2001; Zagórska et al., 2014; Lemke, 2019). We have previously implicated Mertk in postnatal clearance of apoptotic RGCs (Anderson et al., 2022), but a role for TAM receptors in viable RGC elimination was undefined. Here we address whether TAM receptors together with CR3 drive microglial-mediated elimination of embryonic viable RGCs and identify potential cues targeting RGCs for elimination.

## Materials and methods

### Mouse strains

C57BL/6J wild-type mice were obtained from The Jackson Laboratory. B6.129-*Mertk^tm1Grl^*/J and B6.129-*Axl^tm1Grl^*/J strains were a gift from Dr. Greg Lemke and B6.129S4-*Itgam^tm1Myd^*/J strain (Cd11b) was from Jackson laboratory (JAX:003991). Double *Mertk/Axl* and *Mertk/Cr3(Cd11b)* knockouts were generated in house. B6. *Cx3cr1-Gfp/*+ mice were a gift from Richard Lang with permission from Steffen Jung. Mice were housed in an Association for Assessment and Accreditation of Laboratory Animal Care accredited animal facility with 12 h light/12 h dark cycle and *ad libitum* access to food and water. Timed matings were used to determine embryonic age and both sexes were used for all experiments.

### Tissue processing

Newborn pups (P0) were euthanized with isoflurane followed by decapitation. For collection of embryonic tissue, dams were euthanized with isoflurane followed by cervical dislocation. Embryos were carefully removed and dissected. For retinal whole mounts, eyes (e14.5 to P0) were enucleated, and retinas were carefully dissected in ice-cold sterile PBS. Retinas were fixed with 4% PFA for 25–45 min at room temperature while rocking followed by 3 × 10 min washes in PBS. When preparing whole mount retinas for *in situ* hybridization chain reaction (HCR), RNase free conditions were used and retinas underwent serial dehydration (25, 50, 75, 100%) and were stored at −80 °C.

For collection of tissue for cryosectioning, whole heads were fixed in 4% PFA for 45 min and then washed three times in ice-cold sterile PBS for 15 min each followed by consecutive treatments in 10 and 20% sucrose in PBS at 4 °C. Heads were then embedded in OCT compound (Tissue-Tek) by flash freeze in dry ice and 2-Propanol, stored at −80 °C and sectioned at 16 to 50 μm thickness. For *ex vivo* culture, whole eye globes were carefully dissected and incubated in 5 μM PSvue643 reagent (Molecular Targeting Technologies, Inc. Cat#: P-1006) in hibernate medium (Transnetyx, Cat#: HAPR100) for 30 min at ~37 °C, followed by fixation in 4% PFA for 45 min for whole mount retina processing or OCT embedding and sectioning at 16 to 50 μm thickness.

### Immunohistochemistry

Frozen sections were baked at 37 °C for 1 h and dehydrated in ice-cold PBS for 10 min. Sections were washed in PBST (0.1 M PBS and 0.1% Tween 20) for 5 min. If antigen retrieval was required, slides were incubated in sodium citrate buffer for 45 min at >90 °C, and then cooled at room temp for 20 min. Slides were then rinsed 3 times in PBS followed by 0.5–1% triton X for 10 min and 10 min with PBST. Sections were blocked for 2 h at room temperature (0.2% Triton-X, 10% BSA, 10% normal donkey serum in 0.01 M PBS), then incubated in primary antibody overnight at 4 °C (0.2% Triton A, 5% BSA in 0.01 M PBS). The following day sections were washed 3 times in PBST and then incubated with secondary antibodies (5% BSA in PBS) for 2 h at room temperature, washed and mounted with Fluoroshield mounting medium. For whole mount retina immunostaining, retinas were incubated in blocking buffer for 2 h at room temperature (0.2% triton-X, 10% BSA, 10% normal donkey serum in 0.01 M PBS), and subsequently incubated in primary antibody for 3 days at 4 °C (0.2% triton-X, 5% BSA in 0.01 M PBS). For staining of nuclear proteins, an additional step using 50% and then 100% MeOH/PBST was performed prior to primary antibody incubation. Retinas were then washed three times with PBST and incubated with secondary antibodies (5% BSA in 0.01 M PBS) for 2 h at room temperature, proceeded by washes in PBST, PBS rinses and Hoechst staining (1:1000 in PBS) for 25 min, washes and finally mounted with Fluoroshield mounting medium. Antibody information is provided in Table 1.

**Table 1:** Key resources.

Reagent type (species) or resource	Designation	Source or reference	Dilution	Identifiers
Strain, strain background (*Mus musculus*, M/F)	B6.129-*Mertk^tm1Grl^/*J	G. Lemke		10227296
Strain, strain background (*Mus musculus*, M/F)	B6.129-*Axl^tm1Grl^/*J	G. Lemke		10227296
Strain, strain background (*Mus musculus*, M/F)	*Cx3cr1-Gfp/*+	S. Yung		10805752
Strain, strain background (*Mus musculus*, M/F)	B6.129S4-*Itgam^tm1Myd^*/J	JAX		JAX:003991
Antibody	(Rabbit polyclonal) anti-RBPMS	Novus Biologicals	1:1,000	NBP2-20112
Antibody	(Guinea Pig monoclonal) anti-IBA1	Synaptic Systems	1:1,000	234308
Antibody	(Rabbit monoclonal) anti-C1q	Abcam	1:1,500	AB182451
Antibody	Rabbit anti p-cJUN	Cell Signaling	1:500	9261S
Antibody	Mouse anti Brn3a	Millipore	1:250	MAB1585
Antibody	Goat Anti-Brn3b	Rockland	1:200	600-101-MJ0
Antibody	647 (Donkey polyclonal) anti-rabbit	Invitrogen	1:400	A-315573
Antibody	555 (Donkey polyclonal) anti-rabbit	Invitrogen	1:400	A32794
Antibody	488 (Donkey polyclonal) anti-mouse	Invitrogen	1:400	A21202
Antibody	555 (Donkey polyclonal) anti-mouse	Invitrogen	1:400	A31570
Antibody	647 (Donkey polyclonal) anti-mouse	Invitrogen	1:400	A21447
Antibody	555 (Donkey anti Goat)	Invitrogen	1:400	A32816
Antibody	647 (Donkey anti Goat)	Invitrogen	1:400	A21447
Commercial assay or kit	PSvue-643	Molecular Targets	5 nM	P-1006
Commercial assay or kit	*In situ* hybridization chain reaction v3.0 (HCR)	Molecular Instruments (Los Angeles, CA)	Probes 5 nM	https://www.molecularinstruments.com/

### mRNA and immunohistochemistry hybridization chain reaction

For detection of mRNA on sectioned frozen tissue, we followed the manufacturer’s guidelines (Molecular Technologies http://www.moleculartechnologies.org/supp/HCRv2_protocol_generic_solution.pdf) (Choi et al., 2018). In brief, retinas and or sections were prepared in RNase free conditions and stored in methanol or on slides at −80 °C. Upon *in situ* staining, samples were rehydrated serially from methanol to PBST and then incubated in 10 μg/mL proteinase K for 2 min at room temperature followed by incubation with 250 μL probe hybridization buffer for 1 h at 37 °C in a humidified chamber. Retinas were incubated with probes (*C1qb* and *Brn3a*) at 37 °C in a humidified chamber overnight. The following day, probes were washed in probe wash buffer and incubated with hairpins (H1 and H2) at room temperature overnight. The next day, samples were washed in 100% 5X SSCT followed by Hoechst (1:1,000) in PBS, washes in PBS and mounted with antifade mounting reagent (Thermofisher, Cat#: P36930). For HCR- immunohistochemistry, we followed the manufacturer’s guidelines^1^ (Schwarzkopf et al., 2021). In brief, retinas or sections were blocked with antibody buffer and then incubated with primary antibody overnight at 4 °C. The following day, retinas were washed and incubated with secondary antibody with initiator labeled secondary antibodies for 2 h at room temperature, and then hairpins in amplification buffer overnight in dark conditions. The next day, retinas were washed in SSCT and mounted. For detection of protein and mRNA by HCR, methods were merged, by first incubating with primary and secondary antibodies, probes and then amplified using hairpins.

### Confocal microscopy

Images were acquired on an inverted Nikon A1R Confocal Microscope at 20× objective with a 3× digital zoom. Multipoint images were stitched with a 10% overlap. Images of retinal whole mounts were ~100 multipoint images to obtain the entire dorsal retina and sections to obtain the entire coronal eye section. Stacks through the *Z* plane were 0.8 μm steps of ~21 μm thickness. Whole mount retina images represent the max projections of the retina, and for the embryonic retina, the entire thickness of the retina was imaged. For sections, the entire thickness of the section was imaged (~16–20 μm).

### Image analysis

For quantification of RBPMS + RGCs in P0 retinal whole mounts, three ROIs of 0.05 mm^2^ (540 px × 540 px) of the central (0.6 μm to 1.2 μm from the optic nerve head) dorsal retina were analyzed and averaged. Images consisted of ~21 steps at 0.8 μm. For p-cJUN positive RGC counts, three ROIs of 0.05 mm^2^ (540 px × 540 px) of the central (0.6 μm to 1.2 μm from the optic nerve head) retina were analyzed and averaged. For C1q counts, the entire section (16 μm) of the retina (3–4 sections from each eye, including both central and peripheral sections) was counted at a depth of ~21 steps at 0.8 μm.

### Bioinformatics

We accessed our previously published bulk sequencing data set GSE123757 to determine whether embryonic retinal microglia express TAM receptors at e16.5 in comparison to retinal microglia from P7 and P60 (Anderson et al., 2019a). We examined Mertk and Tyro3 genes since we previously published that embryonic retinal microglia express Axl (Anderson et al., 2019a). All bar graphs showing FPKM represent the average FPKM values for each condition ± standard error of the mean (SEM). We accessed single-cell RNAseq data GSE118614 to determine if cells other than microglia in the embryonic retina express TAM receptors (Clark et al., 2019).

### Statistical methods

For image analysis, a minimum of 3 samples (biological replicates) were obtained for each genotype/condition. Image data was analyzed using Prism 9 software (GraphPad, La Jolla, CA). All data were tested for normality using four different tests: Anderson–Darling, D’Agostino & Pearson, Shapiro–Wilk, and Kolmogorov–Smirnov test. We used a one-way ANOVA, and tested for heteroscedasticity in groups of three or more by a Brown–Forsythe test. For comparison of two independent groups that were normally distributed, we used an unpaired parametric *t*-test, and Welch’s *t*-test if variances were unequal. For comparison of two independent groups that were not assumed to be normally distributed, we used a non-parametric Mann–Whitney test. Outliers were not excluded. Both sexes were used. Data is presented as the mean and error bars indicate the standard error of the mean (SEM). We used a 95% confidence interval and a *p*-value of <0.05 for rejecting the null hypothesis. Levels of significance are reported as ^*^p < 0.05, ^**^p < 0.01, ^***^p < 0.001, and ^****^p < 0.0001.

## Results

### Microglia use phagocytic receptors Mertk and CR3 to eliminate a subpopulation of RGCs in the embryonic retina

**Figure 1:**
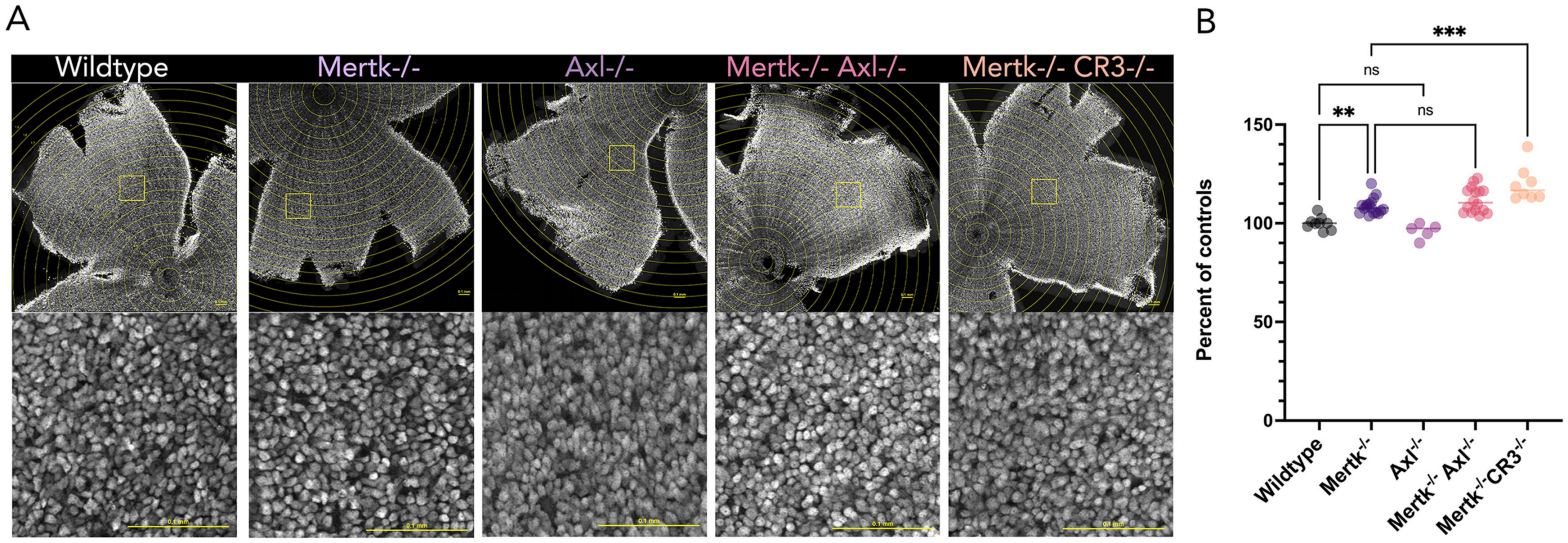
Mertk and CR3 are required for elimination of a subpopulation of embryonic RGCs. **(A)** Whole mount retina images at P0 showing RBPMS-positive (white) RGC density (0.05mm^2^) for the indicated genotypes. Scale bars 0.1 mm. **(B)** Quantification of average RGC density in the dorsal, central retina for the indicated genotypes normalized relative to wild-type control retina and represented as percent of controls. ns, not significant; ^**^p < 0.01; ^***^p < 0.001 Welch’s one-way ANOVA with Brown–Forsythe variance test [points represent average count/eye; *N* = 9 (WT), 17 (Mertk KO), 5 (Axl KO), 16 (Mertk KO Axl KO), 8 (Mertk KO CR3 KO)].

TAM receptors are phagocytic receptors required for efficient removal of apoptotic cells, but their contribution to clearance of viable neurons is not known (Lemke and Burstyn-Cohen, 2010). We previously showed by bulk sequencing analysis of retinal microglia that Axl is expressed embryonically during the period of RGC elimination (Anderson et al., 2019a). To determine whether embryonic retinal microglia express Mertk and Tyro3, we compared developmental expression patterns of retinal microglia from our previously published RNA sequencing datasets (Anderson et al., 2019a). We find that embryonic retinal microglia also express Mertk (Supplementary Figure 1A) but not Tyro3 (not shown). Therefore, we focused on the contribution of Mertk and Axl to engulfment and removal of non-apoptotic RGCs during the embryonic period. We analyzed retinas of Mertk or Axl knockout (KO) mice and wild-type animals for controls (Lemke, 2019; Fourgeaud et al., 2016). These analyses were done at P0/birth since we previously showed that RGCs persist if their phagocytosis is inhibited resulting in a higher density of RGCs (Anderson et al., 2019b). RNA-binding protein with multiple splicing (RBPMS) is a selective marker of RGCs (Rodriguez et al., 2014), and used here to determine RGC density following immunostaining of whole mount retinas. Mertk KO retinas showed a significant increase in RGC density (8.3%) relative to WT controls (Figures 1A,B, *p* = 0.004), while there was no significant difference in Axl KO retinas relative to WT controls (Figures 1A,B; *p* = 0.7149). RGC density was also significantly increased in Mertk/Axl double knockout (dKO) retinas (Figures 1A,B, *p* < 0.0001), but this was not significantly different compared to loss of Mertk alone (*p* = 0.5036). Thus, Mertk is the primary TAM receptor responsible for mediating viable RGC elimination by microglia, while Axl does not contribute.

We previously showed that CR3 is important for elimination of viable RGCs by microglia (Anderson et al., 2019b). To determine whether Mertk and CR3 work together to regulate microglial phagocytosis of RGCs we generated Mertk/CR3 dKO mice. Retinas from these mice showed an increased density of RGCs (21.2%) at birth compared to control (Figure 1B; *p* < 0.0001) and this was significantly increased over loss of Mertk alone (Figure 1B; *p* = 0.0003). This is also a greater increase than our previously reported 10% increase from CR3 KO alone, and comparable to what we observed following microglia depletion (20% increase in RGC density at birth) (Anderson et al., 2019b). To confirm that these receptors are predominetly expressed by microglia and not other cell types in embryonic retina, we analyzed published retinal scRNA-sequencing data for expression of Mertk and Axl during embryonic stages (e14 to e18). We find very low to no expression of Mertk and Axl in other retinal cell types during embryonic stages (Supplementary Figure 1B) (Clark et al., 2019). Collectively, these findings indicate that both Mertk and CR3 are necessary for microglia to efficiently phagocytose viable embryonic RGCs.

### A subpopulation of non-apoptotic RGCs activate c-JUN signaling

**Figure 2:**
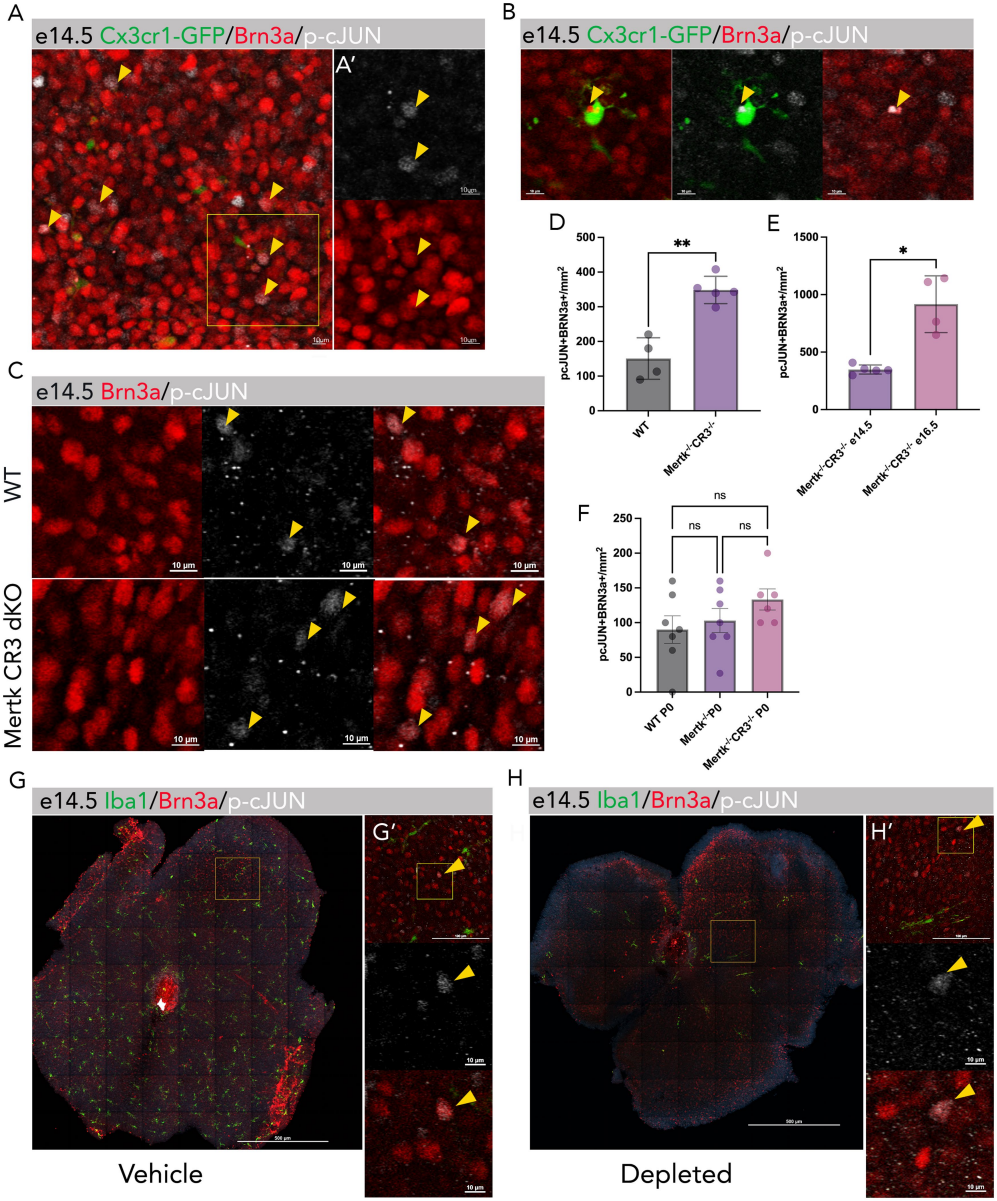
A subpopulation of RGCs express the stress marker phosphorylated c-JUN (p-cJUN). **(A)** Whole mount image (*Cx3cr1-Gfp/+*) of e14.5 retina showing that a subset of RGCs (Brn3a+; red) express nuclear p-cJUN (white; yellow arrows). Boxed regions show enlarged region and label for p-cJUN alone or Brn3a alone **(A′)**. Scale bar = 10 μm. **(B)** Whole mount image (e14.5 *Cx3cr1-Gfp/+*) showing microglia (GFP) internalizing an RGC (Brn3a + red) labeled with p-cJUN (white) using HCR IHC. Scale bar = 1 μm. **(C)** Whole mount images showing RGCs (Brn3a+; red) with nuclear p-cJUN (white) in control mice (WT) and Mertk/CR3 dKO mice. Yellow arrows indicate double positive p-cJUN and Brn3a staining. **(D)** Quantification of double positive p-cJUN + Brn3a + RGCs per mm^2^ in e14.5 control and Mertk/CR3 dKO mice. ^***^p < 0.01 unpaired *t*-test [*N* = 4 (WT), 5 (Mertk CR3 dKO)]. **(E)** Quantification of p-cJUN + Brn3a + RGCs per mm^2^ in e14.5 and e16.5 Mertk/CR3 dKO mice. ^*^p < 0.05 Welch’s *t*-test [*N* = 5 (e14.5), 4 (e16.5)]. **(F)** Quantification of p-cJUN + Brn3a + RGCs per mm^2^ in P0 control, Mertk KO and Mertk/CR3 dKO mice. ns, not significant. Welch’s one-way ANOVA with Brown–Forsythe variance test [*N* = 7 (WT), 7 (Mertk KO), 6 (Mertk CR3 dKO)]. **(G)** Whole mount retina images to show vehicle treated Axl KO mice with RGCs (Brn3a+; red) expressing nuclear p-cJUN (white) and microglia present (IBA1+; green). Scale bar = 500 μm. Boxed regions show enlarged area **(G′)** with yellow arrows indicating double positive p-cJUN and Brn3a staining. Scale bars = 100-10 μm. **(H)** Whole mount retina images to show Axl KO PLX treated mice with depletion of microglia (IBA1; green) and RGCs (Brn3a+; red) with nuclear p-cJUN (white) expression remaining. Boxed regions show enlarged area **(H′)** with yellow arrows indicating double positive p-cJUN and Brn3a staining. Scale bars = 100 to 10 μm.

An unresolved question is why a subset of newborn RGCs are targeted for elimination by microglia. Microglia are responsive to neuronal stress (Frank et al., 2019), raising the question of whether a subset of newborn RGCs may exhibit signs of stress, potentially triggering local microglia to find and eliminate these neurons. The c-Jun transcription factor is activated by phosphorylation in response to cellular stress (Johnson and Nakamura, 2007). By co-immunostaining for phosphorylated c-JUN (p-cJUN) and the RGC marker Brn3a, we found that a subset of embryonic RGCs is labeled for p-cJUN at e14.5 during the peak period of embryonic RGC elimination (Figure 2A). In addition, we show that microglia internalize double positive Brn3a and p-cJUN RGCs (Figure 2B), suggesting this is a potential cue targeting specific RGCs for elimination.

To test the role of microglia in removing p-cJUN positive RGCs, we examined Mertk/CR3 dKO retinas since microglia-mediated RGC elimination is abrogated. At e14.5, we show that there are significantly more p-cJUN positive RGCs (Brn3a+) present in Mertk/CR3 dKO mice (*p* < 0.001; Figures 2C,D) compared to control mice. This suggests that RGCs that express p-cJUN are normally eliminated by a Mertk and/or CR3 dependent mechanism. We next analyzed different timepoints ranging from e14.5 to P0 to determine if p-cJUN + RGCs persisted until birth. From e14.5 to e16.5 there was a progressive increase in p-cJUN labeled RGCs in Mertk/CR3 dKO mice (Figure 2E; *p* < 0.001), suggesting accumulation of stressed RGCs. This resolved by P0 with no significant difference in the number of p-cJUN labeled RGCs between WT, Mertk KO and Mertk/CR3 dKO (Figure 2F). Since there is an increased RGC density at P0 in Mertk/CR3 dKO mice due to disruption of microglia-mediated elimination (Figures 1A,B), this suggests that p-cJUN expression is transient and that RGCs recover and are viable.

### Microglia tag a subpopulation RGCs for elimination

In some contexts, microglia activation can be detrimental to neurons (Marín-Teva et al., 2011), so to distinguish whether microglia stimulate RGC activation of cJUN, we depleted microglia with PLX3397 administration. We used Axl KO mice to allow for greater microglia depletion since we previously showed that Axl is important for survival of a subset of retinal microglia in the absence of CSF1R signaling (Anderson et al., 2022) but itself plays no role in microglia-mediated elimination of RGCs. This method effectively interrupts both pathways, resulting in a substantial depletion of microglia (Figures 2G,H). We found that depletion of microglia in Axl KO mice using PLX3397 still resulted in a subset of RGCs with p-cJUN (Figures 2G′,H′; Supplementary Figure 2). P-cJUN labeled RGCs were not increased following microglia depletion, potentially due to residual clearance activity by the few remaining microglia or that p-cJUN labeled RGCs had not yet significantly accumulated at e14.5. Nevertheless, this suggests that microglia do not induce p-cJUN expression in RGCs, and this mechanism is likely inherent to RGCs.

**Figure 3:**
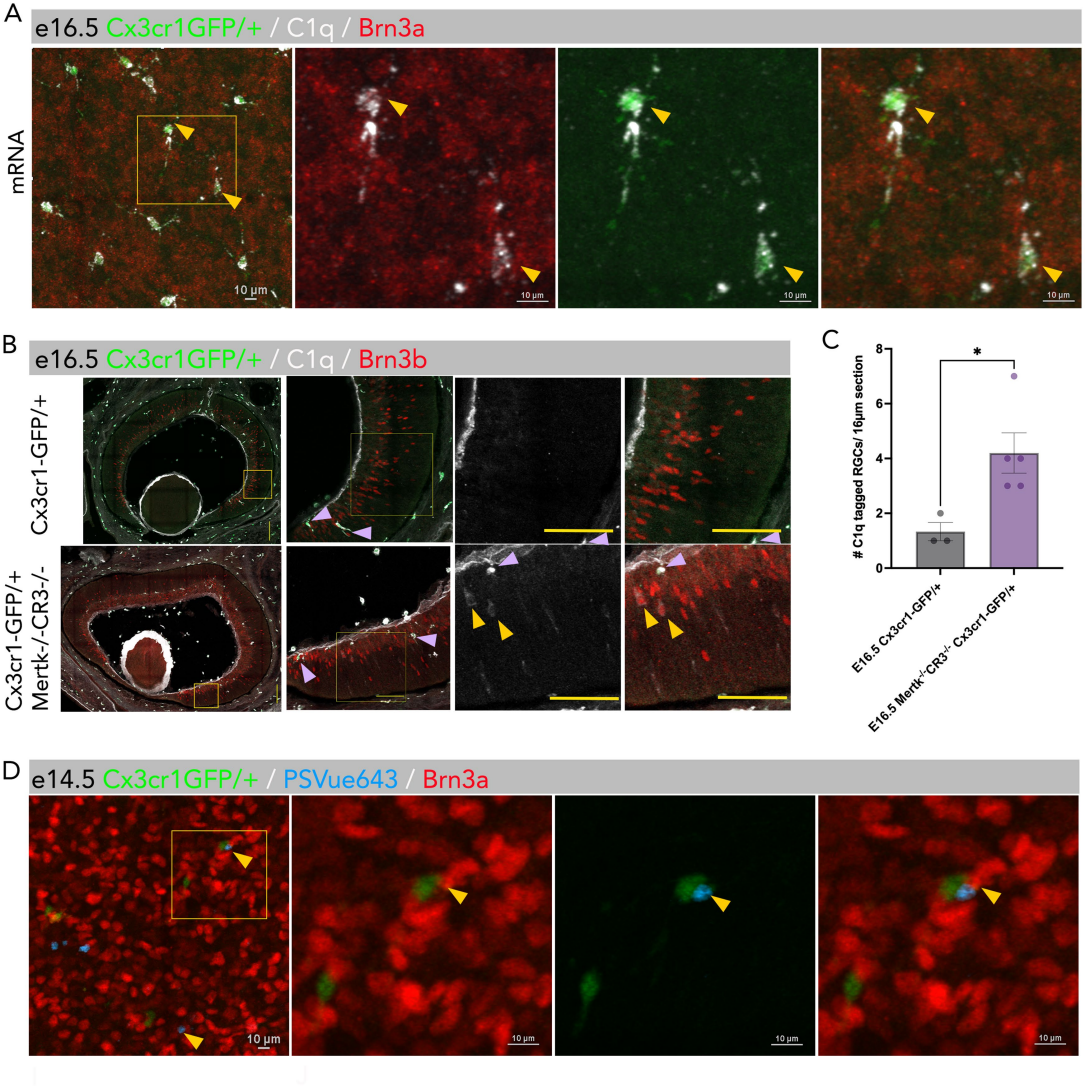
A subpopulation of RGCs display opsonins on their surface. **(A)** Microglia (GFP+) of e16.5 control retinas (*Cx3cr1-gfp/+*) synthesize C1q mRNA (white), whereas RGCs (Brn3a+; red) do not. Boxed regions represent enlarged yellow boxed region in image **A**. Scale bar = 10 μm. *N* = 3. **(B)** Immunostaining for C1q (white) and Brn3b (red) of e16.5 control retinas (*Cx3cr1-gfp/+*) and Mertk CR3 dKO retinas (*Cx3cr1-gfp/*+) showing retention of C1q tagged RGCs (Brn3b+) in mutants. Purple arrows indicate GFP + microglia that express C1q (white) distinct from yellow arrows that indicate Brn3b + cells tagged with C1q (white) and GFP negative [*N* = 3 (WT), 5 (Mertk CR3 dKO)]. **(C)** Quantification of C1q tagged RGCs at e16.5 in control and Mertk CR3 dKO mice. Values represent the average per retina from 3–4 16.5 μm peripheral and central sections. *p* < 0.05 Mann–Whitney test [*N* = 3 (WT), 5 (Mertk CR3 dKO)]. **(D)** *Ex-vivo* culture of e14.5 wild-type mouse eye injected with PSVue643. A subset of RGCs (red) externalize PS (blue), and microglia (GFP+) interact with PS. *N* = 3. Scale bar = 10 μm.

Neurons present a set of signals that can attract or deter microglial phagocytosis (Butler et al., 2021). We have previously shown that a subpopulation of RGCs is tagged with complement protein, C1q (Anderson et al., 2019b), which is considered an “eat me” signal. Here we show that microglia are the only cell type in the embryonic retina that synthesizes C1q mRNA (Figure 3A), suggesting microglia recognize and mark a subset of RGCs for elimination by Mertk and CR3. We reasoned that ablation of Mertk and CR3 may result in excess C1q tagged RGCs due to failure of clearance by microglia. We therefore analyzed mutant retinas at e16.5 and found that loss of Mertk and/or CR3 results in the retention of these C1q tagged RGCs in the retina at e16.5 (Figures 3B,C).

Since Mertk can recognize the eat-me signal phosphatidylserine (PS) (Lemke, 2017), we next analyzed exposure of PS, which is normally restricted to the inner leaflet of the cell membrane. We show that a subset of RGCs expose PS on their surface as seen by PSvue643 labeling (Figure 3D), and we further show that microglia interact with exposed PS on the surface of RGCs (Figure 3D). Thus, multiple cell surface cues and receptor pathways are involved in the recognition and elimination of a subset of RGCs in embryonic retina.

## Discussion

Here we show that microglia eliminate a subset of viable RGCs during development by using both Mertk and CR3 to recognize neurons tagged for elimination, likely due to exposure of C1q and PS. During the embryonic period we find that a subset of RGCs exhibit p-cJUN, indicating transient stress pathway activation, and show that these cells accumulate when microglia-mediated elimination is blocked in Mertk/CR3 KO retinas. These findings establish that microglia selectively clear stressed but potentially viable neurons through specific receptor-mediated recognition, ensuring proper refinement of RGC populations.

Microglia recognize their targets for phagocytosis using specialized receptors that recognize a wide range of signals including molecular patterns, “eat-me” signals and opsonins like C1q (Butler et al., 2021). Our prior work showed that a subset of embryonic RGCs are tagged with complement protein C1q soon after they are generated, and that mice deficient in CR3 fail to eliminate a proportion of RGCs (Anderson et al., 2019b). Since this pathway only partially accounted for the persistence of RGCs after microglia depletion we investigated the role of other phagocytic receptors (Lemke, 2019). TAM receptors, Mertk and Axl are expressed by embryonic retinal microglia and are known phagocytic receptors involved in the removal of apoptotic neurons, but their involvement in elimination of viable neurons was not known (Lemke, 2019; Anderson et al., 2019a). We found that loss of Mertk but not Axl resulted in an excess number of RGCs that persisted until birth (P0), similar to loss of CR3 (Anderson et al., 2019b). We previously observed increased expression of C1q, C3 and Mertk in embryonic retina supporting a central role for these signaling pathways (Anderson et al., 2019b). Disruption of both Mertk and Axl, did not show any cooperativity or redundancy, establishing that Mertk was the primary receptor in addition to CR3 involved in elimination of embryonic RGCs. We showed that loss of both Mertk and CR3 resulted in a higher density of excess RGCs at birth. This was comparable to depletion of microglia (Anderson et al., 2019b), suggesting that they are the principal microglial phagocytic receptors working together to refine RGC number in the embryonic retina. Notably, these findings align with what we previously observed for apoptotic cell recognition by microglia, where we showed that CR3 and Mertk, but not Axl, mediate clearance of apoptotic RGCs in the first postnatal week (Anderson et al., 2022). This reveals that shared pathways are involved in both viable and apoptotic neuron elimination. Given the importance of CR3 and Mertk both developmentally and during postnatal apoptotic-mediated RGC elimination, it would be important to consider the role of these receptors in the removal of possible stressed retinal neurons in pathology such as glaucoma.

Whether a cell is phagocytosed by microglia or not is determined by exposure of signals such as C1q, which is detected by the receptor CR3, or PS, which is recognized by Mertk. Our prior work established a role for C1q (Anderson et al., 2019b), and here we show that microglia interact with exposed PS on the surface of RGCs. Exposure of PS is typically thought to occur during apoptosis (Nagata et al., 2016), but as we reported previously there is negligible apoptosis of RGCs in the embryonic retina (Anderson et al., 2019b). In addition, evidence shows that viable cells can also expose PS transiently and potentially lead to phagocytosis (Segawa et al., 2011; Neher et al., 2013; Tyurina et al., 2007). PS may potentially serve as a scaffold for C1q (Païdassi et al., 2008), and we speculate that this would allow for coordinated tagging and recognition of RGCs by both CR3 and Mertk as we observe. It is also possible that viable RGCs that externalize PS could bind other opsonins to promote microglia-mediated elimination. In Mertk/CR3 dKO mice we observe accumulation of C1q tagged RGCs confirming that they are normally targeted for elimination by microglia through these receptor pathways. It is unclear why two receptor pathways are involved in the removal of embryonic RGCs. They may target RGCs tagged with different eat-me cues, may promote different steps in the engulfment process, or may offer redundancy to ensure unfit RGCs are removed. Complement is upregulated in various contexts of pathology, including glaucoma, which is a progressive degenerative disease that affects RGCs and their axons (Howell et al., 2011). Complement protein, C1q has been implicated tagging and removing unwanted RGC synapses in multiple contexts, including early in glaucoma (Stevens et al., 2007), suggesting a redeployment of microglial-RGC interaction pathways in diverse contexts.

An unanswered question is what leads to RGCs becoming vulnerable and targeted by microglia. The c-Jun N-terminal kinase (JNK) pathway is activated in response to stress (Johnson and Nakamura, 2007), and we show here that p-cJUN, a component of the JNK pathway, is expressed in a subset of Brn3a + RGC nuclei, suggesting that a subset of RGCs are stressed, potentially leading to their removal. This is consistent with externalized PS that we observed on a subset of RGCs during the peak period of embryonic RGC clearance, which is also associated with cellular stress. We further show that mice deficient in Mertk and CR3, fail to remove these p-cJUN expressing RGCs, and they accumulate in the embryonic retina. However, it is also possible that global knockout of key phagocytic receptors or persistence of excess RGCs can induce additional RGC stress and increased activation of p-cJUN. This is a transient event, and we find that p-cJUN levels are normalized by birth while excess RGCs remain in the retina, further suggesting that these RGCs are transiently stressed, and while they are normally removed by microglia, they are viable.

The source of cellular stress for these newborn RGCs is unclear. Several developmental events occur soon after RGCs are generated, including migration of RGCs from the apical surface of the retina, growth of RGC axons, axon fasciculation and axon guidance. It is possible that microglia eliminate stressed or less metabolically fit RGCs before they become a liability to compete for space and synaptic connections. Phagocytic elimination of viable cells is an important quality control mechanism in other contexts as well. In zebrafish, macrophages phagocytose newly formed living stem cells as part of a surveillance process to ensure that only healthy, high-quality hematopoietic stem cells are selected to establish lifelong blood production (Wattrus et al., 2022). Elimination of viable cells has increasingly gained interest and although it is largely appreciated as a method of cell clearance, cell death by phagocytosis may be one of the earliest forms of cell death to have evolved (Brown, 2024).

Altogether, we find that a subset of newborn RGCs exhibit signs of stress by upregulating p-cJUN expression and also present “eat-me” signals to cue microglia for their elimination. We find this is cooperatively dependent on microglial phagocytotic receptors, Mertk and CR3 signaling. This is a normal developmental process to regulate the density of RGCs; however, it is reasonable to think that similar mechanisms could regulate elimination of other cell populations during development or prematurely eliminate stressed, viable neurons in disease (Zhao et al., 2015; Alawieh et al., 2018; Cunningham et al., 2013; Deivasigamani et al., 2023; Neher et al., 2013).

## Data Availability

The raw data supporting the conclusions of this article will be made available by the authors, without undue reservation.

## Footnotes

1
https://files.molecularinstruments.com/HCR™-Gold-RNAFISH-IF-User-Guide.pdf

## Supplementary material

SUPPLEMENTARY FIGURE 1 Embryonic microglia primarily express TAM receptors Mertk and Axl. **(A)** Bar graph of RNA sequencing data for retinal microglia at e16.5, P7 and P30 showing that microglia from developing retina express Mertk. Data from GSE123757 (Anderson et al., 2019a). **(B)** Dot plot visualization of single-cell RNA-sequencing data showing cell types present in developing retina from e14–e18 showing that they do not express Mertk or Axl. Microglia are rare and not present in this dataset. For comparison, RGCs are shown to express high levels of Rbpms and Pouf41 at these stages. Data from GSE118614 (Clark et al., 2019).

SUPPLEMENTARY FIGURE 2 Microglia do not induce p-cJUN expression in RGCs at embryonic stages. Bar graph of p-cJUN + Brn3a + RGCs counts/mm^2^. There is no statistical significance in number of p-cJUN + RGCs between vehicle treated Axl KO mice compared to PLX treated AXL KO mice at e14.5 [unpaired *t*-test with Welch’s correction, *p* = 0.9167; *N* = 7 (Axl KO vehicle), *N* = 3 (Axl KO PLX)].
