# Supplementary figures and images for: Microglial mechanisms of viable retinal ganglion cell elimination
Source: Front Cell Neurosci. 2025 Dec 5;19:1719791. doi: 10.3389/fncel.2025.1719791 (PMC12715010; doi:10.3389/fncel.2025.1719791)

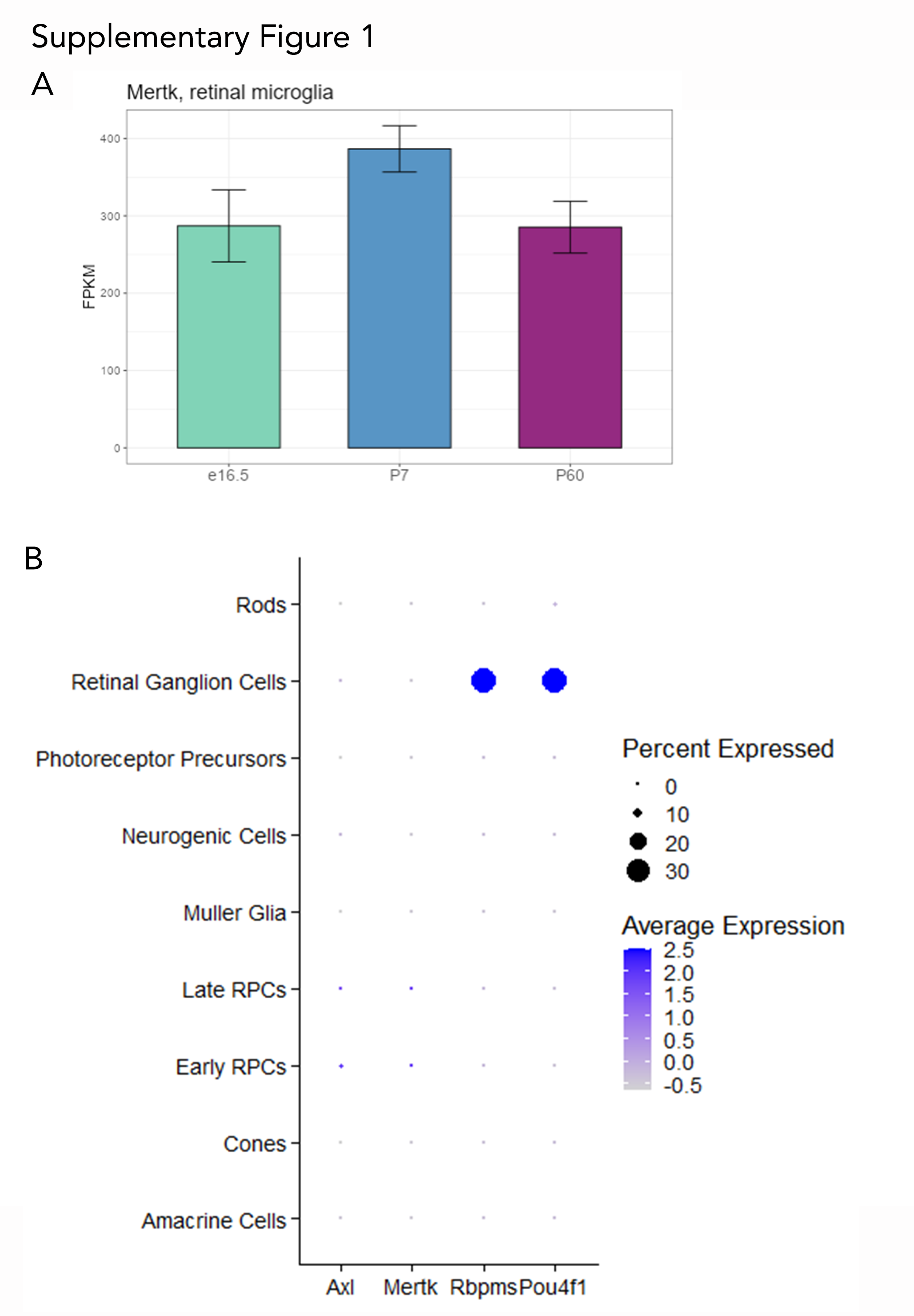

Supplement: SUPPLEMENTARY FIGURE 1 — Embryonic microglia primarily express TAM receptors Mertk and Axl. (A) Bar graph of RNA sequencing data for retinal microglia at e16.5, P7 and P30 showing that microglia from developing retina express Mertk. Data from GSE123757 (Anderson et al., 2019a). (B) Dot plot visualization of single-cell RNA-sequencing data showing cell types present in developing retina from e14–e18 showing that they do not express Mertk or Axl. Microglia are rare and not present in this dataset. For comparison, RGCs are shown to express high levels of Rbpms and Pouf41 at these stages. Data from GSE118614 (Clark et al., 2019). [file Image_1.tif]

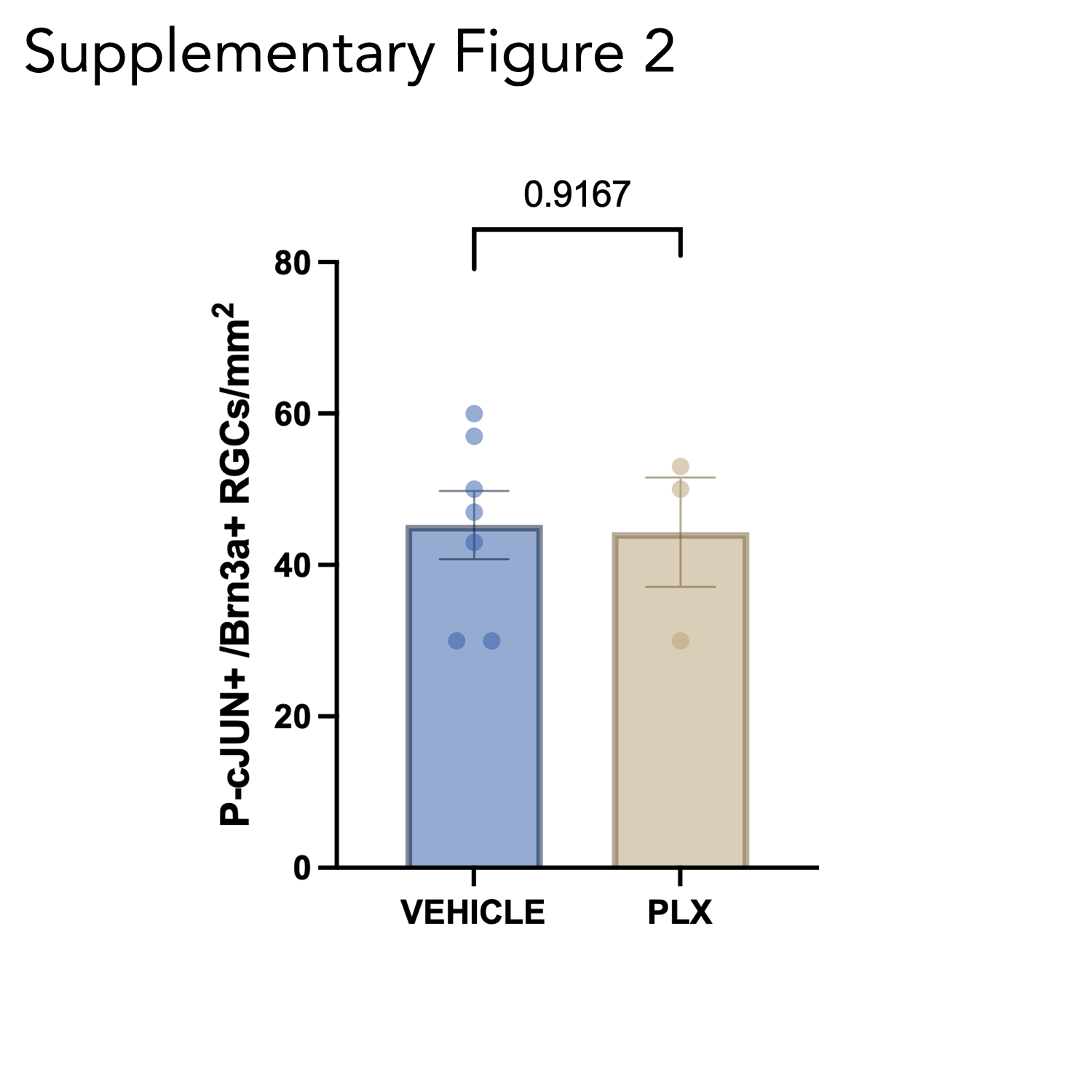

Supplement: SUPPLEMENTARY FIGURE 2 — Microglia do not induce p-cJUN expression in RGCs at embryonic stages. Bar graph of p-cJUN + Brn3a + RGCs counts/mm2. There is no statistical significance in number of p-cJUN + RGCs between vehicle treated Axl KO mice compared to PLX treated AXL KO mice at e14.5 [unpaired t-test with Welch’s correction, p = 0.9167; N = 7 (Axl KO vehicle), N = 3 (Axl KO PLX)]. [file Image_2.tif]
